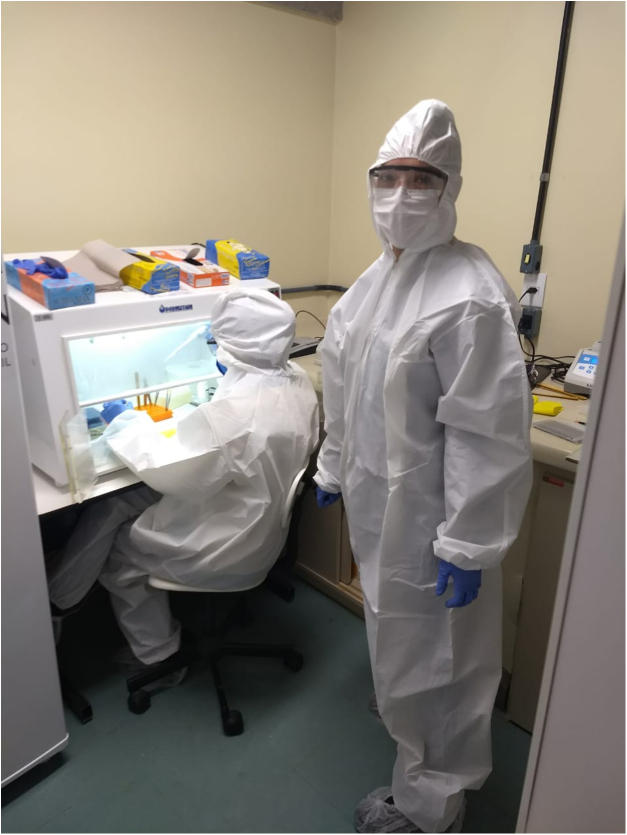# Comparison and optimization for DNA extraction of archived fish specimens

**DOI:** 10.1016/j.mex.2019.06.001

**Published:** 2019-06-08

**Authors:** Priscilla C. Silva, Maria Claudia Malabarba, Richard Vari (In memoriam), Luiz R. Malabarba

**Affiliations:** a Departamento de Zoologia and Programa de Pós-Graduação em Biologia Animal, Universidade Federal do Rio Grande do Sul, Av. Bento Gonçalves 9500, 91.501-970 Porto Alegre, RS, Brazil; b Departamento de Biologia Animal and Programa de Pós-Graduação em Biologia Animal, Universidade Federal de Viçosa, Av. P.H. Rolfs, s/n, 36570-000 Viçosa, MG, Brazil; c National Museum of Natural History, SI, Washington DC, USA

**Keywords:** DNA extraction for museum fish specimens, Ancient DNA, Sanger methodology, Neotropical fish, Museum specimens, Characidae

## Abstract

The DNA extracted from museum alcohol-fixed specimens can be a valuable source of information for solving taxonomic, phylogenetic, ecological and conservational questions. However, this type of DNA, also called ancient DNA, is routinely obtained in small portions and highly fragmented. We have tested two different extraction kits in museum type-specimens of the fish family Characidae. Aiming to increase the DNA yield, we made modifications on a Qiagen manufacturer protocol, in the elution step. Also, to overcome the issue of DNA fragmentation, we applied our efforts in Sanger sequencing, to find a highly variable and, in result, informative COI fragment. Based on our results, there is no correlation between amount of the DNA extracted and the age of the sample. The Sanger sequencing generated sequences which are useful in solving taxonomic puzzles. Here are presented the customization and guidelines that allowed us to recover DNA from the archived fish specimens.

• DNA extraction from archived fish specimens is more effective when using silica columns.
• Change of the elution times from minutes in room temperature to 24 h in freezer greatly improved the DNA yielded.
• Short but highly variable sequences replace the need to sequence the entire gene to identify a species.

## Graphical abstract

**Figure:**
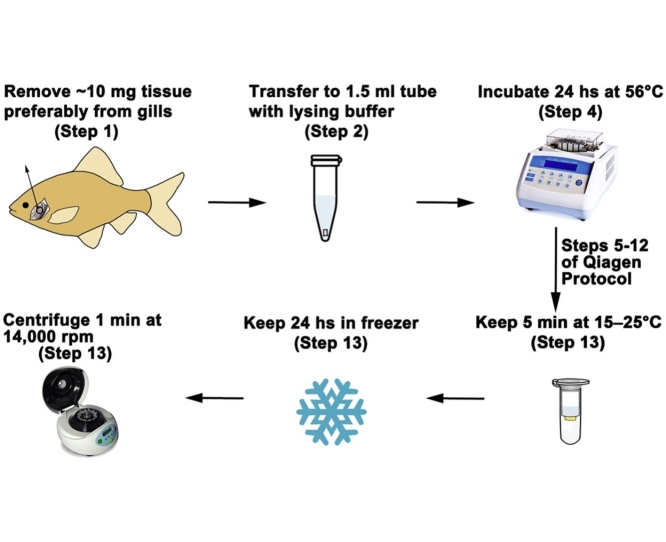

**Specifications Table**

**Table:** 

Subject Area:	Biochemistry, Genetics and Molecular Biology
More specific subject area:	Molecular Systematics and Phylogeny
Method name:	DNA extraction for museum fish specimens
Name and reference of original method:	Our method is a modified version of the Isolation of Genomic DNA from Tissues Protocol protocol for QIAamp® DNA Micro Kit, originally developed by Qiagen for DNA extraction from small quantity of tissue, which we adapt to extract DNA from ancient fish specimens preserved in museum collections.
Resource availability:	QiaAmp Micro Kit (Qiagen) First DNA (Gen-Ial) Mega 6 software

## Method details

### Background

Ancient DNA (aDNA) is the DNA isolated from old samples as subfossil bones, mummies, or museum specimens, that were not properly preserved for DNA extraction. As traditional repositories for biological specimens and tissue samples, museum collections are valuable resources for mapping and naming biodiversity. Nowadays, with the possibility of DNA extraction from archived specimens, the museums become potential storehouses for lots of molecular scientific investigations [1,2].

In taxonomy, the use of aDNA has been a powerful tool for solving problems wherein the type specimens, usually very old, no longer preserve diagnostic features for species identification [3,4]. However, the DNA extracted from this kind of sample is usually little and highly fragmented, restricting the success of further applications.

In order to overcome these issues, we tested extraction kits, reagents, and primers to develop a successful DNA extraction protocol from ancient museum samples. This paper reports our experience extracting and amplifying aDNA from 53 type specimens of Characidae fish family described in the eighteenth, nineteenth and twentieth centuries (Table 1), as well as the modifications introduced in the product recommended protocols that resulted in a successful method to extract DNA from old museum fish specimens.

**Table 1:** List of the sampled type-specimens with quantifications of DNA yield at 5 min, 24 hs and 48 hs. Abbreviations: ANSP = Academy of Natural Sciences of Philadelphia (USA), BMNH = British Museum of Natural History (UK), CAS = California Academy of Sciences (USA), FMNH = Field Museum of Natural History (USA), MCZ = Museum of Comparative Zoology (USA), NMW = Naturhistorisches Museum Wien (AU), UCMZ = University of Cambridge Museum of Zoology (UK), USNM = National Museum of Natural History (USA), ZMUC = Zoological Museum, University of Copenhagen (DZ).

Specimen	Type status	Catalog number	Description year	Extraction ng/ul		
				5 min	24 hs	48 hs
*Astyanax giton*	Lectotype	MCZ 20936	1908	1.0		
*Deuterodon pedri*	Lectotype	MCZ 21081	1908	0.42		
*Deuterodon pedri*	Paralectotype	MCZ 170510	1908			
*Astyanax brevirhinus*	Holotype	MCZ 20905	1908	0.10		
*Astyanax janeiroensis*	Holotype	MCZ 21057	1908	0.93		
*Deuterodon parahybae*	Syntype	MCZ 20933 A	1908	0.59		
*Deuterodon parahybae*	Syntype	MCZ 20933 B	1908	0.64		
*Astyanax scabripinnis intermedius*	Lectotype	MCZ 20684	1908	3.04		
*Astyanax scabripinnis intermedius*	Paralectotype	MCZ 20635	1908	0.16		
*Astyanax scabripinnis intermedius*	Paralectotype	MCZ 20919 A	1908	0.10		
*Astyanax scabripinnis intermedius*	Paralectotype	MCZ 20919 B	1908	0.13		
*Tetragonopterus rutilus jequitinhonhae*	Syntype	NMW 57759	1877	1.38	3.46	0.940
*Tetragonopterus rutilus jequitinhonhae*	Syntype	NMW 57760:1	1877	0.74		
*Tetragonopterus rutilus jequitinhonhae*	Syntype	NMW 57760:2	1877	0.79		
*Tetragonopterus jenynsii*	Syntype	NMW 57534:1	1877	0.69		
*Tetragonopterus jenynsii*	Syntype	NMW 57534:3	1877	0.94		
*Tetragonopterus jenynsii*	Syntype	NMW 57535:1	1877	0.92		
*Astyanax bahiensis*	Syntype	NMW 57251:1	1877	0.76	3.16	0.727
*Astyanax bahiensis*	Syntype	NMW 57252	1877	1.08		
*Tetragonopterus rivularis*	Syntype	USNM 44960 S	1875	0.25		
*Tetragonopterus rivularis*	Syntype	USNM 44960 B	1875	0.60		
*Tetragonopterus rivularis*	Syntype	NMW 57707:1	1875	1.23	2.76	0.227
*Tetragonopterus rivularis*	Syntype	NMW 57708:1	1875	0.92		
*Tetragonopterus rivularis*	Syntype	ZMUC 2074411 P.241372	1875	0.06		
*Tetragonopterus rivularis*	Syntype	ZMUC 2074411 P.241376	1875	1.52		
*Hemigrammus santae*	Syntype	USNM 55652 B	1907	1.14		
*Hemigrammus santae*	Syntype	USNM 55652 S	1907	0.793	2.98	1.01
*Salmo bimaculatus*	Syntype	BMNH 1853.11.12.34	1758	7.0		
*Astyanax bimaculatus novae*	Cotype	FMNH 54641 A	1911	1.61		
*Astyanax bimaculatus novae*	Cotype	FMNH 54641 F	1911	0.910		
*Tetragonopterus jacuhiensis*	Lectotype	ANSP 21912	1894	20.0		
*Tetragonopterus lacustris*	Syntype	NMW 57540	1875	0.44		
*Tetragonopterus lacustris*	Syntype	ZMUC 382 P. 241322	1875			
*Astyanax fasciatus parahybae*	Paralectotype	USNM 120245 1	1908	2.87		
*Astyanax fasciatus parahybae*	Paralectotype	USNM 120245 2	1908	1.27		
*Astyanax fasciatus parahybae*	Lectotype	MCZ 20685	1908	0.14		
*Astyanax fasciatus parahybae*	Paralectotype	MCZ 20891	1908	0.61		
*Astyanax fasciatus parahybae*	Paralectotype	MCZ 20890	1908	0.29		
*Tetragonopterus curvieri*	Syntype	ZMUC P. 241294	1875	1.87		
*Tetragonopterus mexicanus*	Syntype	ZMUC P. 241247	1853	2.01		
*Cheirodon ribeiroi*	Holotype	CAS 59778	1907	1.35		
*Cheirodon ribeiroi*	Paratype	CAS 59779	1907	0.96		
*Hyphessobrycon luetkenii*	Paralectotype	BMNH 1886.3.15.35	1887	2.11		
*Hyphessobrycon luetkenii*	Lectotype	BMNH 1886.3.15.80	1887	2.65		
*Probolodus heterostomus*	Paratype	FMNH 54329	1911	1.58		
*Tetragonopterus taeniatus*	Syntype	UCMZ F.6975.2	1842	0.31		
*Tetragonopterus fasciatus longirostris*	Syntype	NMW 57508	1907	1.03		
*Tetragonopterus laticeps*	Holotype	ANSP 21852	1894	20.0		
*Deuterodon potaroensis*	Paralectotype	FMNH 52968	1909	2.21		
*Tetragonopterus scabripinnis*	Holotype	BMNH 1917.7.14.15	1842	1.19	1.71	0.434
*Astyanax scabripinnis paranae*	Holotype	CAS 22555	1914	4.89		
*Astyanax ribeirae*	Paratype	FMNH 54726	1911	1.87		
*Tetragonopterus eigenmanniorum*	Holotype	ANSP 21598	1894	0.52		

Since aDNA is typically scarce and fragmented, any modern DNA contamination, no matter how small, prevails over the ancient DNA and ends up aborting the results. Therefore, all procedures involved in obtaining and amplifying aDNA were performed following the established sterilization guidelines [[5], [6], [7]], to discard any possibility of contamination.

### DNA extraction

**Fig. 1:**
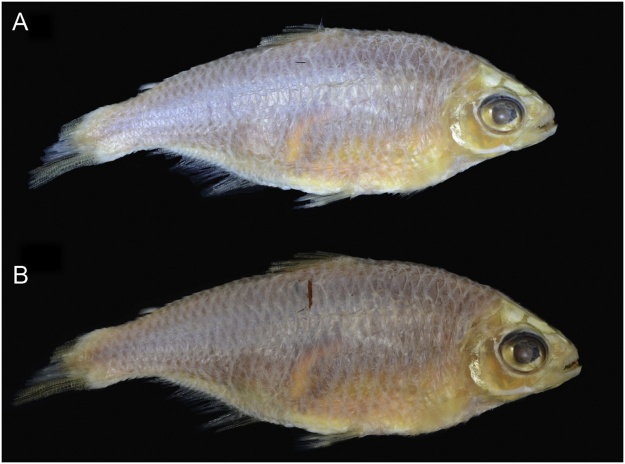
Right side of the lectotype of *Deuterodon pedri* (MCZ 21081) before the incision (A) and after the incision (B), exemplifying position and size of the incision.

We were authorized to sample 53 type-specimens of the Characidae fish family, preserved in different museum collections (see Table 1). Tissue removal was made in a sterile manner and the least invasive possible way to avoid both unnecessary damage to the specimen and contamination of the samples. For this, preferably, part of the branchial arch was removed, otherwise muscle was removed by a very small incision below the dorsal fin, always on the right side of the specimen (Fig. 1), and then immediately inserted in alcohol absolute and cold stored. The tissue was removed in sufficient quantity (30–50 mg) for three DNA extractions, allowing repetition of the process [6].

To avoid contamination with modern DNA, all procedures involving aDNA were performed under maximum cleaning and sterilization conditions, in an isolated and dedicated room [[5], [6], [7]]. The “clean” laboratory, ARCHGEN (Supl. Data 1), has *“one-way” rule of movement*, which means that all reagents only move from this Pre-PCR room to the Post-PCR facilities. As it is required for ancient DNA, the ARCHGEN was created in a room that was never used for manipulating DNA before, and all equipments and disposables were bought strictly for utilizing with ancient samples in there. Basic rules are rigorously, such as: the mandatory use of nitrile gloves, disposable hair caps and shoe covers, respiratory masks, glasses, and polypropylene coveralls for working inside (Supl. Data 2); laboratory personnel cannot reenter after have entered any building in which are any PCR produts. Two kits for DNA extraction were tested: First DNA (Gen-Ial) and QIAamp® DNA Micro Kit. Both kits were used under their manufacturer’s recommendations (For QIAamp® we use QIAGEN Isolation of Genomic DNA from Tissues Protocol). After the extraction, DNA yield was quantitated using the Quantus Fluorometer with QuantiFluor® dsDNA System (Promega) under manufacturer guidelines.Although the DNA quantitation using spectrophotometer have been reported [8,9], is known now that fluorometers are more accurate and accepted [[10], [11], [12], [13], [14]]. According to Rohland and Hofreiteter [11], "Measuring DNA concentration via absorption of UV light at 260 nm may not be sensitive enough; therefore, measurements using fluorescent dyes such as Pico Green, which binds to dsDNA and increases the fluorescent signal, and extrapolation via a standard curve are recommended."

Although both extraction kits showed the presence of DNA in agarose gel, only the Qiagen kit, which uses silica columns, produced viable sequences. We considered a viable sequence those with high quality chromatograms and that the search in BLAST points to the expected species, assuring that the sequences are neither human nor environmental contaminants [7].

Sequences generated from three samples (NMW57759, NMW57760-2 and NMW57540) extracted with the Gen-Ial kit (without silica columns) showed an intense noise and weak signal preventing the reading. Nevertheless, the amplification and sequencing of these same samples, when extracted with the QIAamp, were successful, resulting in viable sequences. We conclude from this that the use of silica columns during extraction results in a cleaner material and free of impurities DNA (PCR and sequencing inhibitors, tissue remains, protein, RNA and extremely small DNA fragments), improving the amplification and the sequencing processes.

**Fig. 2:**
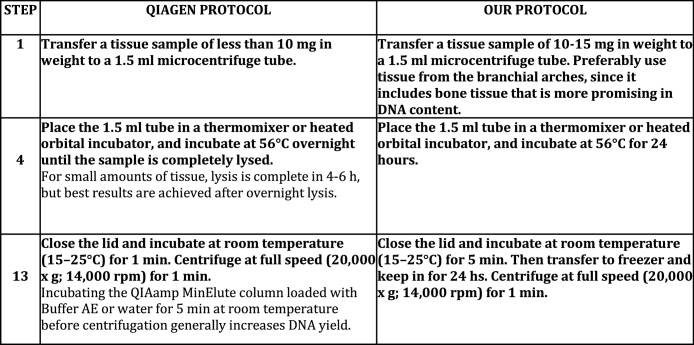
Chart showing the two steps of the DNA extraction with Qiagen protocol (QIAamp® DNA Micro- Isolation of Genomic DNA from Tissues Protocol) which were modified to increase the DNA yield (our).

All extractions resulted positive for presence of DNA, but in variable quantities (Table 1). In order to increase that amount, we carried out tests with the Qiagen QiaAmp Protocol, and we were able to greatly increase the amount of DNA. Remarkably, we noticed that changing the final step of the protocol, passing the time of elution from 5 min in room temperature (the first column of "Extraction" on Table 1) to 24 h in freezer, greatly increased the amount of extracted DNA, even as a second elution (Table 1). Meanwhile, a third elution maintained for 48 h in the freezer showed a decrease in the amount of DNA (Table 1). In Fig. 2 (Fig. 2), we show these steps as they appear in the original Qiagen kit protocol, and in the modified format of our study.

### DNA amplification

Although the NexGen technology (Next Generation sequencing) explores better the fragmentary characteristic of the aDNA, the Sanger technique has a much lower cost, easy use and allows a better control of a given marker, in our case the COI (Cytochrome Oxidase I). Then, it is possible to find a sequence from type specimens that can be used to recognize modern populations of the species for further studies (i.e., phylogeny, ecology).

Our choice of amplifying and sequencing the COI gene was based on its widespread use and availability in public databases including GenBank (https://www.ncbi.nlm.nih.gov/genbank/) and BOLD (http://www.barcodinglife.org/). To overcome the problem of DNA fragmentation, we designed 5 sets of primers (COI-1, COI-2, COI-3, COI-4 and COI-5; Table 2) to amplify small sections of 150–200 bp, which combined would recover the entire COI gene (600 bp). Primer designing was based on an alignment including 217 COI sequences (mean of 600 bp) belonging to 29 Characidae species (Supl. Data 3), attempting to sample the maximum of variability of the specimens at the occurrence area. For building those primer sets we used the tool Oligo Explorer 1.4 (Gene Link, Hawthorne, NY), and checked out their quality and potential efficiency at Oligo Analyzer 1.0.2 [15].

**Table 2:** COI DNA primers designed for this study and their respective high and low melting temperature used in each PCR.

	Primer Sequence Left	Primer Sequence Right	High Melting temperature	Low Melting temperature
COI - 1	5’ GTATTYGTTGCCTGAGCYGG 3’	5’ TATRACRAARGCATGTGCGG 3’	58 °C	56 °C
COI - 2	5’ WTCCCTTTTAGGTGAYGACC 3’	5’ KGGRGGAAGAAGYCARAAGC 3’	56 °C	54 °C
COI - 3	5’GTRATAATYGGRGGRTTTGG3’	5’CCTARAATTGAAGADACACC3’	53 °C	49 °C
COI - 4	5’GTTTACCCYCCTYTWGCYGG3’	5’ATYCCTGCTGCYAGAACBGG3’	60 °C	56 °C
COI - 5	5’HCCAGCYATTTCRCARTACC3’	5’ARRTGTTGATAAAGRATGGG3’	58 °C	54 °C

Two brands of reagents were tested for PCR reactions: Phire Hot Start Taq polymerase (ThermoFisher Scientific) and Hot Start Master mix (Promega). PCR with Phire Hot Start Taq was carried out in a volume of 20 μl containing: 11.6 μl of H20, 4 μl of 10× reaction buffer, 1 μl of dNTPs (2 mM), 1 μl of each primer (10 μM), 0.4 μl (5 U) of Taq and 1 μl of template DNA.

PCR using Promega Hot Start Master was produced in a total volume of 10 μL, containing: 3.45 μl of H20, 5 μl of Master mix (Promega), 0.15 μl of each primer (10 μM), and 1.25 ul of template DNA. PCR thermal profile was the same for both mixes: 94 °C for 3 min for initial denaturation, followed by 5 cycles at 94 °C for 30 s, high melting temperature (see Table 2) for 40 s, and at 72 °C for 1 min, followed by 55 cycles at 94 °C for 30 s, low melting temperature (see Table 2) for 40 s, extension at 72 °C for 1 min, and a final extension at 72 °C for 10 min.

PCR reactions were loaded to a 1% agarose gel together with KAPA universal ladder (Kapa Biosystem), and the products were purified by the Exosap enzymatic method (25% exonuclease, 25% Shrimp Alkaline Phosphatase and 50% deionized water). Sequences were obtained using the Big-Dye reaction on an ABIPrism 3770 automated sequencer from the LAB at NMNH-SI (Laboratory of Analytical Biology at National Museum of Natural History, Smithsonian, Washington DC), Macrogen (South Korea) and Ludwig-ACTGENE (Brazil).

The COI-1 set primer was used 217 times to amplify DNA (including ancient samples and positive control in amplified reactions), of which 47% (102) was checked for presence of bands in agarose and sequenced. Sequencing worked for 21% (22 samples). COI-2 set was tested in 56 samples and bands were confirmed in 37.5% (21) of them. Sequencing was successful in 90.47% (19) of those samples. COI-3 set amplified 29 samples and bands were observable in 51.72% (15) of them, with the exception of two samples where the sequencing failed. COI-5 set was used in 29 samples, forming bands in 34.48% (10); and successfully sequenced for only 20% (2) of the samples. Despite our efforts to increase the specificity, the COI-4 set always showed double bands in the agarose gel, and no sample was sequenced this set. Then, only the sets COI-1, COI-2, COI-3 and COI-5 were considered efficient to amplify COI fragments in archived characid specimens.

**Fig. 3:**
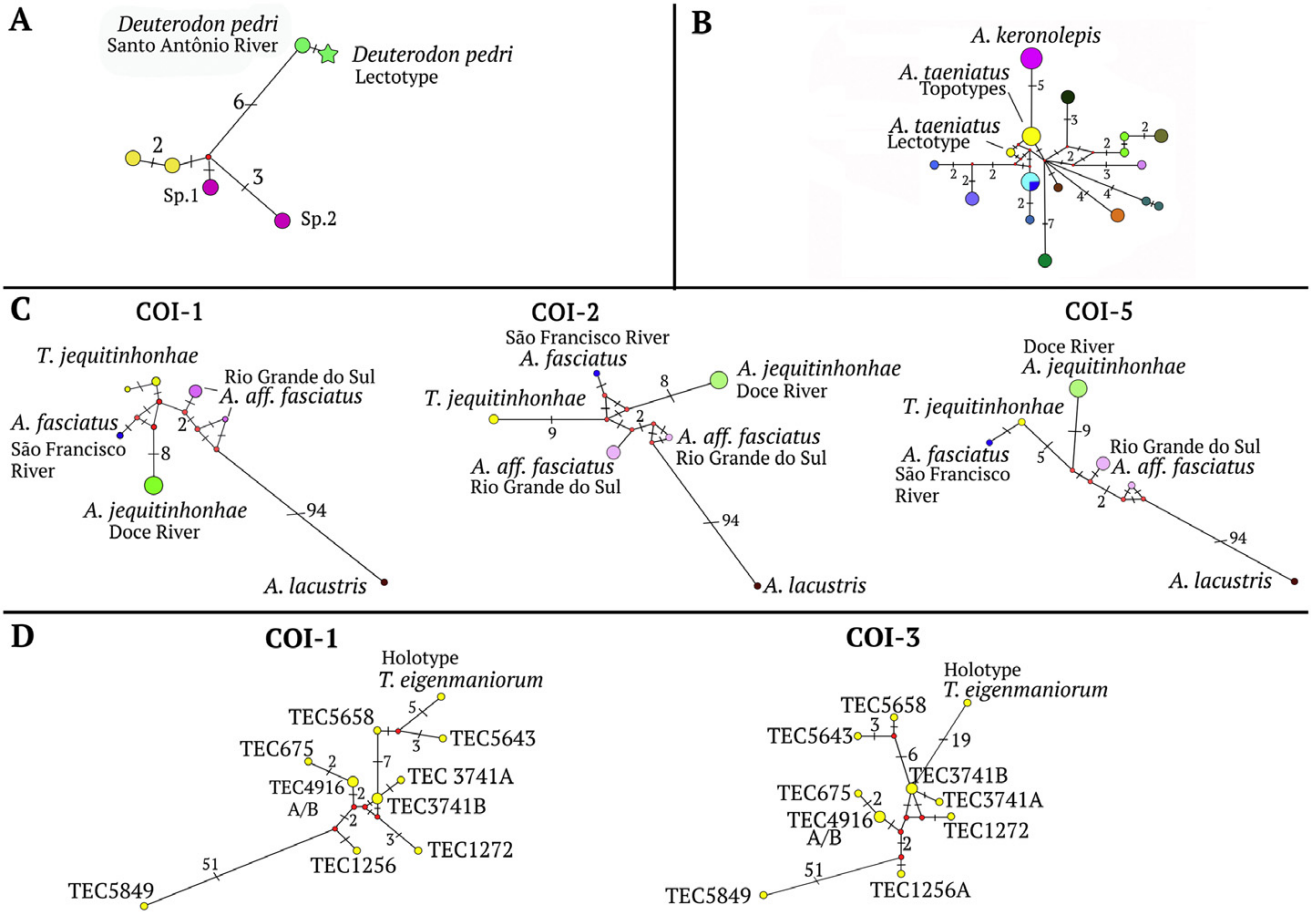
Haplotype networks constructed for sequences from some museum fish specimens and those sequences with low p-distance on the matrix: (A) Haplotype network based on COI-2 of *D. pedri* lectotype (from Silva et al. 2017). (B) Haplotype networks for *Tetragonopterus taeniatus* based on COI-2 showing 5 steps of divergence between this species and *Astyanax keronolepis* (modified from Silva et al. 2019). (C) Haplotype networks for *Tetragonopterus jequitinhonhae*: COI-1 network shows more similarity with *Astyanax fasciatus* from São Francisco river and *Astyanax* aff. *fasciatus* from Rio Grande do Sul. COI-2 network shows a high variability and number of mutational steps (9) between *T. jequitinhonhae* and species with the lowest p-distance on the matriz, indicating absence of a matching sequence. COI-5 network shows more similarity with *Astyanax fasciatus* from São Francisco river. (D) COI-1 and COI-3 network for *Tetragonopterus eigenmaniorum* showing the high number of mutational steps (5 and 19) between the holotype and samples with the lowest p-distance on the matriz. The patterns found in (B) and (C) strongly indicates the absence of a sequence that matches with those of the syntypes (B) and holotype (C).Numbers in each branch refer to number of mutational steps between haplotypes; branches with no number represent only 1 mutational step.

Regarding to variability, COI-1 and COI-5 sets were more conservative than COI-2 and COI-3 fragments (Fig. 3). For example, COI-2 fragment presents 6 mutational steps from the modern population of *Deuterodon pedri* (Fig. 3a) to other species and in *Astyanax taeniatus* where observed 5 mutational steps from other species (Fig. 3b). In the COI-1 and COI-5 fragments, there is only 1 mutational step between *Astyanax rutilus jequitinhonhae* and the remaining samples; whereas in the COI-2 fragment there are 9 steps (Fig. 3c) between them. Also, COI-3 fragment of *Tetragonopterus eigenmaniorum*, 19 mutational steps are counted between this species and remaining samples (Fig. 3d). In short, COI-2 and COI-3 are more variable, and therefore more informative for barcode identifications.

### Negative controls

In both processes, extraction and amplification, we included negative controls for checking contaminations. An extraction negative control, containing no tissue, was processed with each species extraction performed. The quantitation of all negative controls was "lower than blank" meaning that DNA quantity is lower than blank solution used to calibrate the fluorometer.

As regarding to the amplifications, a negative control, containing no DNA, was included at each PCR reaction, which posteriorly were checked in 1% agarose gels.

### Method validation

Our experience reported above demonstrates that even very small archived samples may generate viable DNA sequences. The specimens here studied were collected more than a century ago by naturalists or scientific expeditions in South America, more specifically in Brazil. The Thayer Expedition (1865-1866;), Charles Darwin in the Beagle´s voyage (1832), and Castelnau, as consul of the France in Brazil [[16], [17], [18], [19], [20]], collected specimens which later were used to describe new species. Since these collections occurred before the advent of formalin as fixative, these first naturalists usually fixed the specimens putting them in jars with spirits as rum, brandy, Brazilian cachaça, or whisky [21,22]. As spirits are essentially alcohol, that fixation certainly collaborated to make it possible to obtain viable DNA from such an old material [23].

Although both extraction kits here tested quantified positively for DNA in the spectrophotometer, only the Qiagen kit, which uses silica columns, produced viable sequences. The sequences generated from those samples extracted with the Gen-Ial kit (without silica columns) showed an intense noise and weak signal preventing the reading. Then, we conclude that the use of silica columns in the extraction produces a better quality DNA free of impurities (such as PCR and sequencing inhibitors, tissue remains, and extremely small DNA fragments), improving the amplification and the sequencing processes.

**Fig. 4:**
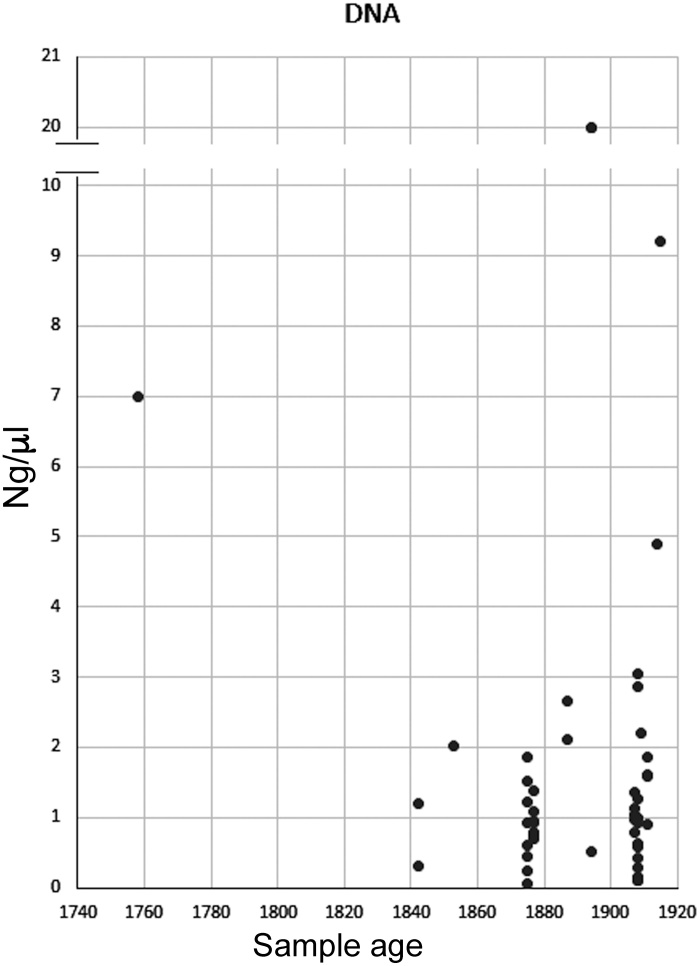
Correlation between concentration of DNA extracted and age (year of description) of the samples. The graphic shows that there is no correlation between these two variables.

Regarding to DNA yielded obtainded with Qiagen kit, no correlation was detected between the amount of DNA extracted and the age of the sample (Fig. 4). As the precise year of specimen collection is not always available, in this study we consider the year of the original description of the species as the age of the sample. However, we must emphasize that collection and fixation precedes, sometimes for several years, the description, as in *A. taeniatus* [24], whose material was collected in 1832 by Darwin, and only 10 years later was described by Jenyns (1842). Instead, we believe that maybe the amount and quality of the extracted DNA is more related with the history and storage conditions of which the specimens were exposed to (i.e., alcoholic degree at fixation, number of specimens fixed together, evaporation, dehydration). As a viable sequence appears to be dependent of the fragmentation degree of the DNA, a good quantity of DNA in the sample it is not a guarantee that the amplification and sequencing processes will succeed.

Both PCR amplification kits, Phire Hot Start Taq polymerase and Hot Start Master mix, worked very well, suggesting that the success of the PCR is dependent on the extracted DNA quality. Thus, the extraction process is the critical step when working with ancient samples.

## Figures and Tables

**Figure:**
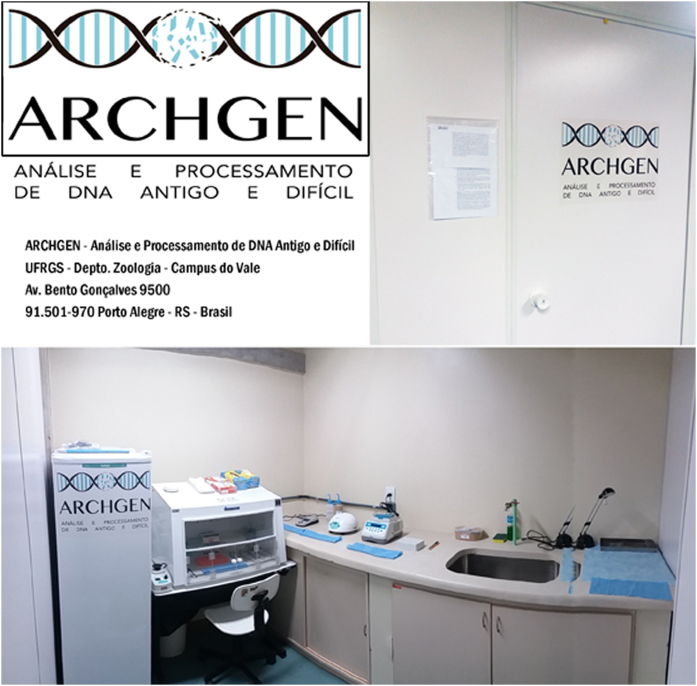


**Figure:**