# Supplementary material for: Comparison and optimization for DNA extraction of archived fish specimens
Source: MethodsX. 2019 Jun 8;6:1433–42. doi: 10.1016/j.mex.2019.06.001 (PMC6584492; doi:10.1016/j.mex.2019.06.001)
Supplement: Supplementary file 1 [file mmc3.docx]

**Supplementary data 1. Pre-PCR laboratory:** ARCHGEN, Ancient DNA Laboratório in the Universidade Federal do Rio Grande do Sul (UFRGS), Porto Alegre, Brazil.

**Supplementary data 2.** Laboratory personnel dressed for working in ARCHGEN with nitrile gloves, disposable hair caps and shoe covers, respiratory masks, glasses, and polypropylene coveralls.

**Supplementary data 3.** Species used for primer design with the accession number of COI sequences deposited at Genbank.
