# Supplementary material for: Comparison and optimization for DNA extraction of archived fish specimens
Source: MethodsX. 2019 Jun 8;6:1433–42. doi: 10.1016/j.mex.2019.06.001 (PMC6584492; doi:10.1016/j.mex.2019.06.001)
Supplement: Supplementary file 2 [file mmc4.doc]

| **Voucher** | **Species** | **Sample number** | **Locality** | **Genbank acession number** |
| --- | --- | --- | --- | --- |
| UFRGS 18508 | *Deuterodon langei* | TEC4103 | Paranaguá River basin | KY327419 |
| UFRGS 18525 | *Deuterodon iguape* | TEC 4138 | Ribeira do Iguape River basin | KY327420 |
| UFRGS 20032 | *Deuterodon iguape* | TEC 4130 | Ribeira do Iguape River basin | KY327421 |
| UFRGS 18495 | *Deuterodon suparis* | TEC 4651 | Itajaí River basin | KY327422 |
| UFRGS 18518 | *Deuterodon singularis* | TEC4087 | Tubarão River basin | KY327423 |
| UFRGS 16519 | *Deuterodon stigmaturus* | TEC2847 | Rio Três Forquilhas | KY327424 |
| UFRGS 16208 | *Deuterodon stigmaturus* | TEC2350 | Maquiné River basin | KY327425 |
| UFRGS 18629 | *Deuterodon langei* | TEC3935 | Cubatão River basin | KY327426 |
| UFRGS18913 | *Astyanax jenynsii* | TEC4271 | Paraíba do Sul River basin | KY327427 |
| UFRGS 17542 | *Astyanax michroschemos* | CT1936 | Doce River basin | KY327428 |
| UFRGS 17542 | *Astyanax michroschemos* | CT1940 | Doce River basin | KY327429 |
| UFRGS19058 | *Astyanax giton* | TEC4033 | Doce River basin | KY327430 |
| MZUFV 4459 | *Astyanax giton* | CT3464 | Doce River basin | KY327431 |
| MZUFV 4458 | *Astyanax intermedius* | CT2801 | Doce River basin | KY327432 |
| UFRGS18894 | *Astyanax intermedius* | TEC4554 | São João River basin | KY327433 |
| MCP 47661 | *Deuterodon pedri* | CT2521 | Doce River basin | KY327434 |
| UFRGS17543 | *Deuterodon pedri* | CT2529 | Doce River basin | KY327435 |
| MZUFV3992 | Sp1 | CT2353 | Doce River basin | KY327436 |
| MZUFV3992 | Sp1 | CT2765 | Doce River basin | KY327437 |
| MZUFV 4457 | Sp2 | CT2965 | Doce River basin | KY327438 |
| MZUFV 4457 | Sp2 | CT2971 | Doce River basin | KY327439 |
| UFRGS18957 | *Astyanax lacustris* | TEC4772 | Santa Maria da Vitória River basin | KY327440 |
| UFRGS19055 | *Astyanax lacustris* | TEC4030 | Tiririca lake, Doce River basin | KY327441 |
| UFRGS 18503 | *Astyanax laticeps* | TEC4113 | Ribeira de Iguapé River basin | KY327442 |
| UFRGS 18503 | *Astyanax laticeps* | TEC4115 | Ribeira de Iguapé River basin | KY327443 |
| MZUFV 4456 | *Astyanax scabripinnis* | CT2772 | Doce River basin | KY327444 |
| MZUFV 4456 | *Astyanax scabripinnis* | CT2773 | Doce River basin | KY327445 |
| UFRGS19070 | *Astyanax* aff.  *fasciatus* | TEC4074 | Doce River basin | KY327446 |
| UFRGS19746 | *Astyanax* N sp | TEC5291 | Tripuí river, Doce River basin | KY327447 |
| UFRGS 19147 | *Astyanax fasciatus* | TEC4865A | Tramandaí River basin | KY327448 |
| UFRGS 19147 | *Astyanax fasciatus* | TEC4865 B | Tramandaí River basin | KY327449 |
| UFRGS 19135 | *Astyanax fasciatus* | TEC4853A | Tramandaí River basin | KY327450 |
| UFRGS 19135 | *Astyanax fasciatus* | TEC4853B | Tramandaí River basin | KY327451 |
| UFBA 07798 | *Myxiops aphos* | A | Paraguaçu drainage | KY327452 |
| UFBA 07798 | *Myxiops aphos* | B | Paraguaçu drainage | KY327453 |
| ROM96089 | *Jupiaba essequibensis* | T15810 | Essequibo River, Guyana | KY327454 |
| ROM96166 | *Jupiaba mucronata* | T16213 | Guyana | KY327455 |
| UFRGS18758 | *Probolodus heterostomus* | TEC4184 | Paraíbuna River, Paraíba do Sul River basin | KY327456 |
| UFRGS22004 | *Serrapinus heterodon* | TEC6956 | Doce River basin | KY327457 |
| UFRGS18431 | *Hyphessobrycon luetkenii* | TEC3824 | Maquiné River, Tramandaí River basin | KY327458 |
| UFRGS19226 | *Hyphessobrycon luetkenii* | TEC4921 | Mostardas River | KY327459 |
| UFRGS 19342 | *Astyanax taeniatus* | TEC4997 | Macaé River basin | KY327460 |
| UFRGS 19342 | *Astyanax taeniatus* | TEC5000 | Macaé River basin | KY327461 |
| UFRGS 18516 | *Astyanax ribeirae* | TEC 4112 | Ribeira do Iguape River basin | KY327462 |
| UFRGS 20032 | *Astyanax ribeirae* | TEC 4137 | Ribeira do Iguape River basin | KY327463 |
| UFRGS 18904 | *Astyanax hastatus* | TEC 4527 | Macaé River basin | KY327464 |
| UFRGS 18906 | *Astyanax hastatus* | TEC 4529 | Macaé River basin | KY327465 |
| UFRGS 18773 | *Astyanax keronolepis* | TEC4192 | Ubatumirim River basin | MK012668 |
| UFRGS 18795 | *Astyanax keronolepis* | TEC4206 | Taquari River basin | MK012669 |
| UFRGS 18795 | *Astyanax keronolepis* | TEC4208 | Taquari River basin | MK012670 |
| UFRGS 18797 | *Astyanax keronolepis* | TEC4210 | Mambucaba River basin | MK012671 |
| UFRGS 18822 | *Astyanax keronolepis* | TEC4220 | Guapimirim River basin | MK012672 |
| UFRGS 18860 | *Astyanax keronolepis* | TEC4228 | Guapimirim River basin | MK012673 |
| UFRGS 18860 | *Astyanax keronolepis* | TEC4233 | Guapimirim River basin | MK012674 |

UFRGS = Universidade Federal do Rio Grande do Sul; MCP = Museu de ciência e tecnologia da Pontifícia Universidade Católica do Rio Grande do Sul; MCZ= Museum of comparative zoology of Harvard University; MZUFV = Museu de zoologia João Mojeen da Universidade Federal de Viçosa.
